# Analysis of inpatient cost burden and influencing factors of seniors’ patients with mental illness in Dalian, China

**DOI:** 10.1186/s12877-023-04424-w

**Published:** 2023-11-14

**Authors:** Xin Dai, Mingcheng Gao, Yue Liu, Run Lv, Huanhong Chen, Huayi Miao, Ying Zhang

**Affiliations:** School of Public Health, Dalian Medical University, Dalian, China

**Keywords:** Hospitalization costs, Seniors, Mental disorder

## Abstract

**Background:**

As China's aging population continues to grow, the prevalence of mental illness among the seniors has been steadily increasing. The aim of this study is to reveal the changing trends and characteristics of economic burden among seniors patients with long-term hospitalization for mental illness, and to analyze the influencing factors.

**Methods:**

The data for this study were gathered from seniors’ patients with mental illness who were hospitalized and aged 60 years or older. The patients were admitted to four specialized and general hospitals located in Dalian city between January 2018 and December 2020. The types of diseases include affective mental disorders (mood disorders), Schizophrenia, schizotypal, and delusional disorders, Organic (including symptomatic) mental disorders, Neurotic, stress-related and somatoform disorders, Mental retardation, Mental and behavioral disorders due to substance use. (Identify the main diagnosis at discharge using ICD-10 coding). This study analyzed the basic characteristics and disease-related information of seniors patients with long-term psychiatric disorders who were hospitalized, and explored the factors influencing hospitalization costs among patients with different illnesses.

**Results:**

Among the 3871 study subjects, the average length of hospital stay was 127.51 days. The average hospitalization expenses per case were 33,656.07 yuan. Seniors’ patients with mental illness who receives treatment in specialized hospitals have higher hospitalization costs. Long-term hospitalization increases the total hospitalization costs. Age has an impact on hospitalization costs for patients with organic mental disorders. Patients with affective disorders (mood disorders) and neurotic, stress-related, and somatoform disorders who are covered by urban employee medical insurance have higher hospitalization costs.Patients with severe psychiatric disorders who have a 31-day readmission plan, as well as senior patients with somatoform disorders comorbid with other illnesses, incur higher hospitalization costs.

**Conclusions:**

We should take corresponding measures to reduce the number of readmissions for patients with severe mental illnesses. The impact of treatment methods and differences in healthcare institutions on total hospitalization costs deserves further research. It is necessary to strengthen the prevention and diagnosis of comorbid physical illnesses in patients with mental disorders. The burden of mental illnesses in the seniors is significant, and medical insurance policies should be inclined towards providing support.

**Supplementary Information:**

The online version contains supplementary material available at 10.1186/s12877-023-04424-w.

## Background

The impact of mental illness on health has been increasing year by year. Between 1990 and 2019, the reduction in dareability-adjusted life years from the control of communicable diseases, maternal, newborn and nutritional diseases was more than offset by the increased burden of non-communicable diseases such as mental illness [1–3]. According to the latest epidemiological survey, the lifetime prevalence of mental disorders (excluding senile dementia) in China is 16.57%, with a 12-month prevalence rate of approximately 9.32% [4]. The economic burden caused by mental illness accounts for a relatively high proportion of the total global economic burden of all diseases [5]. The inpatient costs of patients with mental illness in the UK account for 22% of the total healthcare service costs, highlighting the significant impact of mental illness on healthcare resources [6]. Mental illness is a major component of the healthcare expenditures in the United States, accounting for approximately $201 billion in 2010 [7]. For seniors patients, although they have Medicare, Medicaid and employer supplements, they still face the burden of paying out-of-pocket medical expense [8–11]. Therefore, researchers believe that individual countries need to place more emphasis on prevention in policy, practice, and research to address the growing burden of mental illness [12, 13].

In recent years, as China's aging population continues to grow [14], the prevalence of mental illness among the seniors has been steadily increasing. This not only impacts the quality of life for the seniors, but also results in a rise in medical expenses for this demographic. As a result, this places a significant economic burden on both patients and their families, as well as society as a whole. Seniors’ patients with mental illness typically exhibits symptoms such as memory loss, abnormal mental behavior and a decrease in daily functional ability. Additionally, they are often characterized by a high risk of relapse and are difficult to cure. Prevention and rehabilitation training should be emphasized in seniors’ patients with mental illness. However, current treatments often rely solely on drug therapy, resulting in longer hospital stays and increased medical costs for this demographic. Hospitalization costs are a significant part of medical expenses for senior patients. Previous studies have identified several factors that affect the cost of hospitalization for seniors’ patients with mental illness, including length of stay, number of physical illnesses, type of medical insurance, hospital where they are located, age, and gender [15].

Accurate data on disease costs are crucial for making informed decisions regarding the allocation of resources [12, 16].The majority of low- and middle-income countries allocate less than 1% of their healthcare budget to mental health, primarily because governments do not give enough attention to mental illness and fail to implement policies in place [17]. In China, studies have been conducted to analyze the cost of hospitalization or outpatient treatment for specific mental disorders, such as depression, schizophrenia, and mood disorders, in different regions. However, there is a lack of analysis of hospitalization costs among older adults with various mental disorders, and there is a lack of studies that address the differences in factors influencing hospitalization costs among patients with different disorder types. The case study area has a significant aging population, and as a result, this study collected and analyzed hospitalization cost information of senior’s patients with mental illness from multiple medical institutions in Dalian City from 2018 to 2020. The aim of this study is to reveal the changing trends and characteristics of economic burden among seniors’ patients with long-term hospitalization for mental illness, and to analyze the influencing factors, provide a basis for the development of prevention and control measures for seniors’ mental illness, and offer a reference for the development of health services and security systems for seniors’ mental illness.

## Methods

This study is based on data obtained from the medical record information centers of psychiatric inpatients in four hospitals in Dalian City. The following criteria were used to determine data inclusion and exclusion: (1) The primary diagnosis is a mental disorder (identified by ICD-10 coding for the main diagnosis at discharge); (2) The discharge date falls between January 1, 2018, and December 31, 2020; (3) The actual hospitalization period is 60 days or more; (4) Patients under 60 years old are excluded; (5) Data with missing information are excluded; and (6) Data beyond three standard deviations from the mean are removed using the Z-score method. (The excluded patients are mostly civil servants, while the remaining patients purchase supplementary private health insurance. According to interview findings, this group of patients often opts for inpatient care instead of home care, resulting in higher medical expenses.) Ultimately, 3871 patient hospitalization records were included in the study.

According to age classification, developed countries define individuals aged 65 and above as seniors, whereas China defines individuals aged 60 and above as seniors. For the purposes of this study, seniors’ patients refer to individuals aged 60 and above who has been diagnosed with a mental illness and were hospitalized in one of four hospitals in Dalian City. The study focuses specifically on seniors’ patients with mental illness who were aged 60 and above.

Firstly, this study analyzed the basic characteristics of seniors’ patients with mental illness, with a focus on their personal characteristics. Secondly, the study examined the disease status of seniors’ patients by analyzing disease-related information of those who were hospitalized for an extended period. Long-term hospitalization is determined based on the criteria of medical insurance payment. DRG (Diagnosis-Related Groups) is a method of categorizing patients into specific groups based on their basic characteristics, disease diagnosis, treatment methods, severity, and other relevant data through the calculation of big data. These groups are subsequently used to determine the weighted fee rates for the settlement of medical insurance funds. Patients with a hospital stay of up to 60 days are eligible for Diagnosis-Related Group (DRG) payment, while stays exceeding 60 days are considered non-acute period diagnoses and are not covered by DRG payment. Therefore, this study includes patients with a hospital stay of 60 days or more as the study subjects for long-term hospitalization. Concurrent complications, such as cardiovascular disease and diabetes, were also analyzed as accompanying symptoms of seniors’ patients with mental illness, based on interviews with clinical psychiatrists. Finally, the study analyzed the influencing factors of hospitalization costs for seniors’ patients with different diseases, based on the differences in hospitalization costs for seniors’ patients with mental illness among various factors.

### Statistical analysis

Descriptive statistical analyses were performed on factors such as patients' basic conditions and disease types, as well as the three-year average sub-hospitalization costs of different categories of patients for each factor. The dependent variable for this study is the hospitalization expenses of patients, while the independent variables include the personal characteristics of senior’s patients with mental illness, disease types and related factors, and insurance types. Variables were selected based on their meanings to analyze the influencing factors, and two-way ANOVA was used to explore the differences in hospitalization expenses among different groups of independent variables for each disease type. For two independent samples that conform to normal distribution but have unequal variances, a corrected t-test was used. For two independent samples that do not conform to normal distribution, the Mann–Whitney U test was used. The Kruskal–Wallis H test was used for three or more groups. Factors that significantly affects hospitalization expenses in the single-factor analysis were included in the multiple linear regression model. The stepwise method was used to analyze the influencing factors of hospitalization expenses for each disease type, and the significance level for entering and excluding variables was set at α = 0.05.

## Results

Among the 3871 study subjects, the average length of hospital stay was 127.51 days (median 104.00, interquartile range 77.00–154.00), and the average age was 68.39 years (median 66.00, interquartile range 63.00–73.00). Among the types of medical insurance, the largest number of patients covered by employee medical insurance is followed by the payment from the new rural cooperative medical insurance. Over the three-year period, hospitalization expenses for patients covered by employee medical insurance payment increased annually. The patients predominantly seek hospitalization in specialized medical facilities, and there has been a consistent upward trend in average hospitalization costs year after year. Conversely, hospitalization expenses for patients hospitalized in comprehensive hospitals decreased year by year.

Over the three-year period, the average hospitalization expenses of patients who did not plan to be readmitted within 31 days after discharge increased annually. The primary diagnosis for patient discharge was affective disorders (mood disorders) (ICD-10: F30-F39), and the hospitalization expenses for this disease increased annually over the three-year period, but the increase in 2020 was relatively small compared to the previous year. The second most common diagnosis was schizophrenia, schizotypal and delusional disorders (ICD-10: F20-F29), and the hospitalization expenses for these diseases decreased significantly between 2018 and 2019 but increased again the following year. The number of cases of mental and behavioral disorders caused by psychoactive substances was the smallest, but the average expenses per case increased almost twofold over the three-year period. The average hospitalization cost per patient over three years was 33,656.07 yuan (with a median of 27,789.79 yuan and an interquartile range of 19,208.48 to 38,424.27 yuan). Specifically, the average hospitalization costs per patient in the years 2018, 2019, and 2020 were 32,088.25-yuan, 34,147.93 yuan, and 34,379.41 yuan, respectively. Table 1 displays the basic information of the patients and the variations in hospitalization expenses. Based on the results of the two-way ANOVA, there are significant differences in the average hospitalization costs per patient between specialized hospitals and general hospitals for different categories of seniors’ psychiatric patients. For most categories, patients in specialized hospitals tend to have higher hospitalization costs. Table 2 presents the impact of hospital category (F1, P1) on hospitalization costs, the influence of different variables in the first column of the table (F2, P2), and the effect of interaction (F3, P3).

**Table 1:** The basic information and changes in hospitalization costs of seniors’ patients with mental disorders from 2018 to 2020

Case situation	Category	Number of cases	Annual average hospitalization cost in 2018 (Yuan)	constituent ratio	Annual average hospitalization cost in 2019 (Yuan)	Constituent ratio	Annual average hospitalization cost in 2020 (Yuan)	Constituent ratio
Age	60 ~ 69	2525	35,743.72	39.13%	33,798.21	32.40%	34,322.56	32.47%
	70 ~ 79	953	27,849.41	30.49%	34,249.47	32.83%	33,249.67	31.46%
	80 ~	393	27,742.41	30.37%	36,277.39	34.77%	38,122.61	36.07%
Gender	Male	1759	35,047.77	54.21%	36,953.69	53.78%	35,408.79	51.35%
	Female	2112	29,605.10	45.79%	31,753.62	46.22%	33,549.76	48.65%
Medical insurance type	Employee medical insurance	2050	26,679.43	15.94%	29,334.72	20.19%	31,823.16	21.49%
	Medical insurance for both urban and rural residents	1313	30,350.79	18.14%	39,222.00	26.99%	34,092.91	23.02%
	Settlement is divided into two times	452	72,948.22	43.60%	38,224.66	26.31%	57,091.59	38.56%
	At one's own expense	56	37,351.64	22.32%	38,512.97	26.51%	25,064.83	16.93%
Hospital category	Specialized hospital	2851	30,102.84	43.77%	36,189.12	56.23%	41,144.98	68.96%
	General hospital	1020	38,666.43	56.23%	28,166.63	43.77%	18,519.41	31.04%
Have a 31-day readmission is scheduled	Yes	173	18,911.31	37.08%	13,606.49	28.47%	11,228.29	22.92%
	No	3698	32,088.25	62.92%	34,190.05	71.53%	37,769.32	77.08%
Disease	Affective mental disorders (mood disorders)(ICD-10:F30-F39)	1624	24,028.91	12.55%	29,002.68	13.34%	29,448.46	11.86%
	Schizophrenia, schizotypal, and delusional disorders(ICD-10:F20-F29)	1269	44,791.63	23.40%	36,113.34	16.62%	40,461.98	16.29%
	Organic (including symptomatic) mental disorders(ICD-10:F00-F09)	628	33,287.73	17.39%	38,376.30	17.66%	31,663.40	12.75%
	Neurotic, stress-related and somatoform disorders(ICD-10:F40-F48)	193	22,825.93	11.93%	29,835.77	13.73%	41,036.79	16.53%
	Mental retardation (ICD-10:F70-F79)	80	35,743.06	18.68%	41,559.57	19.12%	45,351.62	18.26%
	Mental and behavioral disorders due to substance use(ICD-10:F10-F19)	77	30,714.38	16.05%	42,445.99	19.53%	60,369.29	24.31%
Medical insurance payment method	Pay by bed day	934	26,142.4	42.83%	38,767.76	54.15%	12,523.62	23.94%
	Else	2937	34,902.13	57.17%	32,832.02	45.85%	39,787.03	76.06%

**Table 2:** Differences in average hospitalization costs per case among patients of different categories in specialized and general hospitals

Variables	Category	Specialized hospital		General hospital		F_1_	P_1_	F_2_	P_2_	F_3_	P_3_
		Example number	Annual average hospitalization cost (Yuan)	Example number	Annual average hospitalization cost (Yuan)						
Age	60 ~ 69	1784	37,293.15	741	27,701.03	50.134	0.000	2.653	0.000	2.465	0.000
	70 ~ 79	742	33,789.55	211	25,410.15						
	80 ~	325	34,384.57	68	23,776.64						
Gender	Male	1182	40,774.97	577	25,916.73	194.878	0.000	2.224	0.000	74.445	0.000
	Female	1669	32,703.29	443	28,331.53						
Medical insurance type	Employee medical insurance	1463	29,624.57	587	29,570.77	0.320	0.571	83.360	0.000	193.038	0.000
	Medical insurance for both urban and rural residents	905	40,495.43	408	21,793.33						
	Settlement is divided into two times	452	48,726.59								
	At one's own expense	31	24,654.74	25	50,797.31						
Have a 31-day readmission is scheduled	Yes			172	11,154.22	4.776	0.029	2.714	0.074	2.961	0.045
	No	2851	36,051.48	848	30,172.51						
Disease	Affective mental disorders (mood disorders)	1452	28,078.88	172	23,681.91	180.964	0.000	58.822	0.000	18.048	0.000
	Schizophrenia, schizotypal, and delusional disorders	634	49,610.63	635	29,783.44						
	Organic (including symptomatic) mental disorders	459	39,912.46	169	21,614.80						
	Neurotic, stress-related and somatoform disorders	177	35,430.21	16	11,933.28						
	Mental retardation	62	46,577.65	18	21,140.19						
	Mental and behavioral disorders due to substance use	67	46,231.14	10	29,468.86						
Medical insurance payment method	Pay by bed day	649	33,293.56	285	11,639.88	58.247	0.000				
	Else	2202	36,862.08	735	32,908.10						

After the Spearman's correlation test, age had a statistically significant impact on the hospitalization expenses of seniors’ patients with affective disorders, organic mental disorders, neurotic disorders, stress-related disorders, and somatoform disorders. Hospitalization days also had a statistically significant impact on the hospitalization expenses of seniors’ patients with all six types of mental illnesses. Additionally, the number of physical illnesses had a statistically significant impact on the hospitalization expenses of seniors’ patients with affective disorders, neurotic disorders, stress-related disorders, and somatoform disorders.

By the Mann–Whitney U test or the t-test, gender only had a statistically significant impact on the hospitalization expenses of seniors’ patients with affective disorders (mood disorders) among the six types of mental illnesses. The hospital type had a statistically significant impact on the hospitalization expenses of seniors’ patients with all six types of mental illnesses. Additionally, whether there was a plan to be readmitted within 31 days had a statistically significant impact on the hospitalization expenses of seniors’ patients with five types of mental illnesses, except for mental and behavioral disorders caused by psychoactive substances. Moreover, the medical insurance payment method had a statistically significant impact on the hospitalization expenses of seniors’ patients with five types of mental illnesses, except for affective disorders (mood disorders) (Table 3).

**Table 3:** Correlation Analysis of Various Influencing Factors and Inpatient Costs

Variable	Statistical value	Affective mental disorders (mood disorders)inpatient costs	Schizophrenia, schizotypal, and delusional disorders inpatient costs	Organic (including symptomatic) mental disorders inpatient costs	Neurotic stress-related and somatoform disorders inpatient costs	Mental retardation inpatient costs	Mental and behavioral disorders due to substance use inpatient costs
Age	r_s_	0.068^**^	-0.022	-0.111^**^	0.074^**^	0.000	-0.024
	P	0.006	0.432	0.006	0.008	0.998	0.839
Hospitalization days	r_s_	0.815^**^	0.905^**^	0.886^**^	0.876^**^	0.927^**^	0.869^**^
	P	0.000	0.000	0.000	0.000	0.000	0.000
Number of somatic diseases	r_s_	0.063^*^	-0.027	-0.053	0.264^**^	0.019	-0.116
	P	0.011	0.335	0.183	0.000	0.865	0.317

^*^P < 0.05

^**^P < 0.01

By the Kruskal–Wallis H test, the medical insurance type had a statistically significant impact on the hospitalization expenses of seniors’ patients with five types of mental illnesses, except for mental and behavioral disorders caused by psychoactive substances. The Table 4 displays the analysis of Factors Influencing Hospitalization Expenses by Disease Type—Single Factor Analysis.

**Table 4:** Analysis of Inpatient Costs Influencing Factors for Different Disease Types—Univariate Analysis

Variable	Category	Affective mental disorders (mood disorders)			Schizophrenia, schizotypal, and delusional disorders			Organic (including symptomatic) mental disorders			Neurotic, stress-related and somatoform disorders			Mental retardation			Mental and behavioral disorders due to the use of psychoactive substances		
		Cost (YUAN)	Z/H	P	Cost (YUAN)	Z/H	P	Cost (YUAN)	Z/H	P	Cost (YUAN)	t/Z/H	P	Cost (YUAN)	t/Z/H	P	Cost (YUAN)	t/Z/H	P
Gender	Male	29,717.50	-3.01	0.003	40,091.69	-0.136	0.892	35,263.30	-1.079	0.280	36,182.64	0.767	0.444	41,419.83	0.504	0.616	44,529.87	0.613	0.542
	Female	26,664.05			39,281.00			34,441.54			31,883.85			38,679.70			40,867.39		
Medical insurance type	Employee medical insurance	26,166.82	21.165	0.000	35,086.40	86.558	0.000	31,840.19	66.113	0.000	36,656.47	-3.391	0.001	27,951.97	0.670	0.505	33,279.55	7.613	0.055
	Medical insurance for both urban and rural residents	30,272.01			37,401.22			35,603.80			28,887.09			40,509.02			43,325.43		
	Settlement is divided into two times	42,075.08			50,411.85			45,656.09									49,584.34		
	At one's own expense	20,336.38			39,777.94			29,728.14									40,502.21		
Hospital category	Specialized hospital	28,078.88	-3.183	0.001	49,610.63	-13.136	0.000	39,864.26	-10.490	0.000	35,430.21	9.996	0.000	46,577.65	5.499	0.000	46,231.14	2.184	0.032
	General hospital	23,681.91			29,783.44			21,614.80			11,933.28			21,140.19			29,468.86		
Have a 31-day readmission is scheduled	Yes	11,041.66	-7.175	0.000	11,242.09	-14.047	0.000	11,268.14	-11.322	0.000	12,169.07	-4.040	0.000	11,138.19	-5.444	0.000			
	No	27,662.15			41,526.83			37,271.99			34,524.77			46,098.23			44,054.22		
Medical insurance payment method	Pay by bed day	28,325.22	-0.452	0.651	23,822.33	-15.078	0.000	28,907.01	-7.159	0.000	19,005.28	-6.609	0.000	29,881.72	-4.684	0.000	30,550.55	-2.908	0.005
	Else	27,467.41			44,368.34			38,820.82			38,962.86			43,643.25			47,880.26		

For two independent samples that conform to the normal distribution but have unequal variances, a corrected t-test is utilized. In contrast, for two independent samples that do not conform to the normal distribution, the Mann–Whitney U test is employed. When it comes to three or more independent samples, the Kruskal–Wallis H test is utilized as a multiple sample rank test

### Results from the regression analyses

Stepwise multiple linear regression was employed to analyze the factors influencing hospitalization expenses for each of the six types of mental illnesses. The natural logarithm of hospitalization expenses was used as the dependent variable, and factors that had a significant impact on hospitalization expenses in the single-factor analysis were included in the multiple linear regression model as explanatory variables.

After verification, the standardized residuals for all six regressions were found to conform to a normal distribution. The results of the regression models confirmed significant influencing factors, with an adjusted r^2^ = 0.734 for affective disorders (mood disorders); an adjusted r^2^ = 0.886 for schizophrenia, schizotypal, and delusional disorders; an adjusted r^2^ = 0.834 for organic (including symptomatic) mental disorders; an adjusted r^2^ = 0.855 for neurotic, stress-related, and somatoform disorders; an adjusted r^2^ = 0.909 for intellectual disability; and an adjusted r^2^ = 0.823 for mental and behavioral disorders due to psychoactive substance use.

The gradually multiple regression analysis of hospitalization costs for each type of disease is presented in Table 5. Based on the results of the regression analysis, the length of hospital stay was found to be a significant factor affecting all six types of mental illnesses and had the most significant impact on the hospitalization costs of seniors’ patients with different mental illnesses. The hospital category also had a significant impact on the hospitalization costs of seniors’ patients with all six types of mental illnesses. The payment method of medical insurance had a significant impact on the hospitalization costs of seniors’ patients with five types of mental illnesses, except for affective disorders. When compared to other types of medical insurance, employee medical insurance had a more significant impact on the hospitalization costs of seniors’ patients with two types of mental illnesses. Additionally, age only had a significant impact on the hospitalization costs of seniors’ patients with organic (including symptomatic) mental disorders. The presence of a 31-day readmission plan had a significant impact on the hospitalization costs of seniors’ patients with four types of mental illnesses, excluding neurotic, stress-related, somatoform disorders, and mental and behavioral disorders caused by the use of psychoactive substances. Moreover, the number of physical illnesses had a significant impact on the hospitalization costs of seniors’ patients with neurotic, stress-related, and somatoform disorders.

**Table 5:** Analysis of Inpatient Cost Influencing Factors for Various Disease Types—Stepwise Multiple Regression Analysis

Disease category	Variables	Non-standardized coefficients		Standardized regression coefficient(β)	t	P
		B	Std. Error			
Affective mental disorders (mood disorders) r2 = 0.734	Constant	3.865	0.025		153.788	0.000
	Hospital day	0.003	0.000	0.846	63.673	0.000
	Have a 31-day readmission is scheduled	0.181	0.025	0.099	7.172	0.000
	Hospital category	-0.056	0.009	-0.087	-6.448	0.000
	Employee medical insurance	0.019	0.006	-0.042	3.161	0.002
Schizophrenia, schizotypal, and delusional disorders r2 = 0.886	Constant	3.845	0.024		162.744	0.000
	Hospital day	0.002	0.000	0.723	70.413	0.000
	Medical insurance payment method	0.156	0.007	0.249	21.537	0.000
	Hospital category	-0.110	0.006	-0.210	-17.394	0.000
	Have a 31-day readmission is scheduled	0.129	0.013	0.118	10.216	0.000
Organic (including symptomatic) mental disorders r2 = 0.834	Constant	4.144	0.051		80.622	0.000
	Hospital day	0.003	0.000	0.762	44.768	0.000
	Medical insurance payment method	0.093	0.010	0.165	9.310	0.000
	Hospital category	-0.108	0.012	-0.174	-8.935	0.000
	Have a 31-day readmission is scheduled	0.113	0.020	0.117	5.671	0.000
	Age	-0.002	0.001	-0.070	-4.180	0.000
Neurotic, stress-related and somatoform disorders r2 = 0.855	Constant	3.932	0.022		179.381	0.000
	Hospital day	0.003	0.000	0.723	24.536	0.000
	Medical insurance payment method	0.176	0.027	0.289	6.496	0.000
	Hospital category	-0.163	0.031	-0.165	-5.323	0.000
	Number of somatic diseases	0.019	0.004	0.137	4.763	0.000
	Employee medical insurance	0.051	0.023	0.093	2.246	0.026
Mental retardation r2 = 0.909	Constant	3.879	0.101		38.580	0.000
	Hospital day	0.002	0.000	0.730	17.775	0.000
	Medical insurance payment method	0.093	0.027	0.140	3.478	0.001
	Hospital category	-0.132	0.042	-0.178	-3.133	0.002
	Have a 31-day readmission is scheduled	0.113	0.052	0.130	2.179	0.032
Mental and behavioral disorders due to substance use r2 = 0.823	Constant	4.018	0.037		107.348	0.000
	Hospital day	0.003	0.000	0.823	16.929	0.000
	Medical insurance payment method	0.190	0.033	0.281	5.817	0.000
	Hospital category	-0.127	0.041	-0.152	-3.124	0.003

## Discussion

The research findings indicate the following:①Psychiatric patients treated in specialized hospitals incur higher hospitalization costs.②Longer hospital stays lead to increased total hospitalization expenses.③Age influences hospitalization costs for patients with organic mental disorders.④Emotional psychiatric disorders (mood disorders) and neurotic, stress-related, and somatic symptom disorders among seniors patients covered by urban employee medical insurance result in higher hospitalization expenses.⑤Patients with severe psychiatric disorders who have a 31-day readmission plan experience higher hospitalization costs.⑥Patients with somatic symptom disorders who also have comorbid physical illnesses experience increased hospitalization costs. The higher hospitalization costs impose a significant financial burden on families of seniors’ psychiatric patients in China.

The study found that hospital type significantly impacts six types of mental illness in seniors’ patients, with specialized hospitals having higher hospitalization costs than general hospitals. Specialized hospitals have a lower self-payment ratio for medical insurance patients compared to general hospitals. Moreover, in China, mental health services are primarily provided by secondary and tertiary hospitals, with over 90% of these services offered by psychiatric hospitals [18]. Based on interviews with doctors from specialized hospitals, specialized hospitals tend to have a higher percentage of out-of-town patients, and seniors’ out-of-town patients usually have complex medical conditions. They typically seek treatment in specialized hospitals only when they encounter difficulties receiving treatment in their local area [19]. Patients treated in specialized hospitals have more complex medical conditions and longer average hospital stays than those treated in general hospitals, this can result in increased hospitalization costs. Specialized hospitals are increasingly adopting a per-diem payment system. However, under the per-diem payment system for mental illness, hospitals may engage in behaviors that encourage demand for medical insurance outside of coverage, prolong hospitalization days, and increase hospitalization frequency [20]. Similarly, it could lead to higher hospitalization expenses.

Through the analysis of hospitalization costs for seniors’ patients with six types of mental illnesses, it was discovered that the length of hospital stay had the greatest impact on hospitalization costs among all influencing factors. The longer the length of hospital stay, the more medical resources the patient consumes, and the higher the hospitalization costs. This result is consistent with previous research on the correlation between length of hospital stay and hospitalization costs for patients with mental illnesses [10]. Moreover, the impact of length of hospital stay on hospitalization costs did not show any significant change in the seniors’ patient population or in patients who were hospitalized for a long period of time.

This study specifically focuses on seniors’ patients with mental illnesses, and the impact of age on hospitalization costs differs from previous studies on seniors’ patients. The study discovered that age indirectly affects hospitalization costs through the length of hospital stay, and the effect is negative [7]. However, after conducting multiple factor analysis, this study found that age had a significant impact on hospitalization costs for patients with organic (including symptomatic) mental disorders. Specifically, the older the patient with this type of disorder, the higher the hospitalization costs. In this study, the proportion of seniors’ patients with organic mental disorders who have two or more concurrent diseases was 57.80%, which is higher than the average of 48.77% among those with six different diseases. The proportion of patients with three or more concurrent diseases was 28.02%, still higher than the average of 22.24% among those with six different diseases. Organic mental disorders are mental disorders that are secondary to other systemic and brain disorders [21]. The older a person is, the higher the likelihood of having coexisting physical illnesses, and the more likely to have organic mental disorders and higher hospitalization costs after co-morbidities. The relationship between age and hospitalization costs for the other five types of mental illnesses was found to be not significant. This could be due to the fact that the study focused on seniors’ patients who were hospitalized for a long period of time. Based on actual data, the comorbidities for seniors’ patients with long-term psychiatric disorders during hospitalization primarily consist of chronic illnesses such as hypertension and diabetes, and their condition is relatively stable with less medication compared to acute patients. The hospital expenses increase with the extension of hospital stay and are averaged with relatively fewer expenses in other aspects.

When compared to the compensation status of medical insurance for different insured and uninsured patients, the reimbursement and compensation ratios of urban employee medical insurance are the highest [11]. An analysis of hospitalization costs for seniors’ patients with mental illnesses reveals that the proportion of expenses, such as medication, that may not be covered by medical insurance is relatively low. In this study, the proportion of hospitalization costs paid out-of-pocket by retired employees is lower compared to other medical insurance types. The results of multiple factor analysis indicate that employee medical insurance has a significant impact on hospitalization costs for seniors’ patients with affective disorders (mood disorders) and neurotic, stress-related, and somatoform disorders, two types of mental illnesses. Patients who participate in employee medical insurance have higher hospitalization costs. When it comes to the unique characteristics of mental illnesses, they are prone to recurrence and have a prolonged course of illness [22]. The average length of hospital stay for patients with affective disorders (mood disorders) is 104.98 days, while patients with neurotic, stress-related, and somatoform disorders have an average length of hospital stay of 119.27 days, which is higher than the overall average length of hospital stay for the study subjects. The readmission rate for patients with affective mental disorders (mood disorders) is 31.72%, which is relatively high compared to the readmission rate of 16.58% for patients with neurotic, stress-related, and somatoform disorders. Patients with these two types of illnesses require more care and long-term treatment compared to other illnesses. The lower the proportion of hospitalization costs paid out-of-pocket by patients, and the more difficult it is to receive home care, the higher the likelihood of family members and patients being hospitalized for long-term treatment and care, resulting in higher hospitalization costs.

The study found that the presence of a 31-day readmission plan has a significant impact on hospitalization costs for four types of mental illnesses among seniors’ patients. These include affective disorders (mood disorders), schizophrenia, schizotypal and delusional disorders, organic (including symptomatic) mental disorders, and intellectual disabilities. Readmission within a month is closely related to hospitalization costs for patients, with the majority being patients with severe mental illnesses such as schizophrenia and bipolar disorder [10]. Seniors’ patients with severe mental illnesses have lower functional abilities compared to patients in other age groups and require care in medical facilities with complete facilities. As a result, they often require readmission after discharge, which affects the length of hospitalization and indirectly affects hospitalization costs.

Research has found that, after controlling for other variables, comorbidities such as cardiovascular disease, diabetes, and liver disease are significantly linked to increased hospitalization costs for patients [17]. The majority of seniors patients with comorbid physical illnesses in this study had the aforementioned diseases. The research findings revealed a significant correlation between the number of physical illnesses and hospitalization costs for seniors’ patients with neurotic, stress-related, and somatoform disorders. As the number of physical illnesses increased, so did the hospitalization costs. Moreover, physical illnesses were often the primary symptoms of mental disorders. The study found that 91.9% of patients with somatoform disorders had an average of 2.8 Co-occurring mental disorders [23]. Compared to the other five mental disorders, this disease has a higher rate of comorbidity with mental disorders. Additionally, for patients with this disease, the presence of concurrent physical illnesses may result in the emergence of new mental symptoms or worsen pre-existing mental disorders [24]. As a result, this leads to a prolonged hospitalization period, which indirectly results in increased hospitalization costs.

The high cost of hospitalization places a heavy burden on families of senior’s patients with mental illness. To address this issue, China has implemented various cost control and protection measures, such as:(1) Legislating to include mental health funding in the budget, establishing psychiatric or psychological outpatient clinics in comprehensive hospitals, and providing appropriate subsidies to mental health workers can strengthen the prevention of mental disorders;(2) China implemented the eliminating drug markups to reduce hospital drug sales, although public hospitals still increase patient medication through prescriptions and additional service fees through clinical pathways; (3) strengthening hospital cost monitoring by strictly controlling cost indicators through monthly and quarterly monitoring; and (4) implementing a tiered diagnosis and treatment policy where common diseases are treated at local community hospitals and transferred to higher-level hospitals if necessary. The tiered diagnosis and treatment system can reduce the pressure and economic burden on patients seeking medical treatment. Currently, China is promoting the implementation of the tiered diagnosis and treatment system through medical security system reforms.

## Conclusions

We should take corresponding measures to reduce the number of readmissions for patients with severe mental illnesses. The impact of treatment methods and differences in healthcare institutions on total hospitalization costs deserves further research. seniors’ patients with affective disorders (mood disorders) and neurotic, stress-related, and somatoform disorders can be transferred to community hospitals for follow-up care after receiving treatment in high-level hospitals. It is necessary to strengthen the prevention and diagnosis of comorbid physical illnesses in patients with mental disorders. The burden of mental illnesses in the seniors is significant, and medical insurance policies should be inclined towards providing support. Additionally, the government should increase investment in mental health funding to effectively alleviate the burden on patients and society. We hope to continue conducting large-scale follow-up research activities on hospitalization costs for seniors’ patients with mental illnesses in China.

### Strengths

This study collected hospitalization cost data of seniors’ patients with mental illness from multiple medical institutions and effectively utilized the advantages of multi-institutional data to compare and analyze the differences in hospitalization costs between specialized and general hospitals. The study focused on seniors’ individuals with various mental disorders and conducted a differential analysis of the factors affecting hospitalization costs for patients with different types of diseases.

### Limitation

While the data in this study were collected from multiple medical institutions and has a larger sample size and greater representativeness compared to data from a single institution, it still lacks national representativeness. The distribution of resources and service levels for mental health specialty services in China varies greatly, and this study only analyzed hospitals in Dalian. Additionally, although this study maximized the inclusion of information and variables contained in the Homepage of inpatient) medical records, it did not include clinically relevant variables or patient information after discharge. Therefore, the guidance this study provides for clinical practice is relatively limited.

### Supplementary Information

**Additional file 1: Table 1. **Composition of hospitalization expenses for seniors’ patients with mental illness from 2018 to 2020.

**Additional file 2. **Original Data.

## Data Availability

The datasets used and/or analyzed during the current study available from the corresponding author on reasonable request.
