# Supplementary material for: Analysis of inpatient cost burden and influencing factors of seniors’ patients with mental illness in Dalian, China
Source: BMC Geriatr. 2023 Nov 14;23:739. doi: 10.1186/s12877-023-04424-w (PMC10644489; doi:10.1186/s12877-023-04424-w)
Supplement: Supplementary file 1 — Additional file 1: Table 1. Composition of hospitalization expenses for seniors’ patients with mental illness from 2018 to 2020. [file 12877_2023_4424_MOESM1_ESM.docx]

**Additional table 1**

Table 1 Composition of hospitalization expenses for elderly patients with mental illness from 2018 to 2020

| Cost | | Affective mental disorders (mood disorders) | Schizophrenia, schizotypal, and delusional disorders | Organic (including symptomatic) mental disorders | Neurotic, stress-related and somatoform disorders | Mental retardation | Mental and behavioral disorders due to the use of psychoactive substances |
| --- | --- | --- | --- | --- | --- | --- | --- |
| Treatment cost | Sub-average  (yuan) | 13874.44 | 18535.77 | 14526.43 | 16106.29 | 20484.81 | 32267.25 |
|  | Constituent ratio | 46.82% | 45.83% | 45.88% | 48.10% | 50.15% | 53.45% |
| Examination fee | Sub-average  (yuan) | 3295.94 | 4179.35 | 3281.21 | 5569.18 | 6940.7 | 5746.1 |
|  | Constituent ratio | 11.19% | 10.33% | 10.36% | 16.63% | 16.99% | 9.52% |
| Bed charge | Sub-average  (yuan) | 3375.77 | 4713.54 | 3628.35 | 2895.22 | 3625.86 | 5853.75 |
|  | Constituent ratio | 11.47% | 11.65% | 11.46% | 8.65% | 8.88% | 9.70% |
| Nursing costs | Sub-average  (yuan) | 3166.57 | 4559.82 | 3629.66 | 2900.7 | 3940.58 | 5599.62 |
|  | Constituent ratio | 10.75% | 11.27% | 11.46% | 8.66% | 9.65% | 9.28% |
| Western medicine cost | Sub-average  (yuan) | 2606.96 | 2431.92 | 2876.85 | 2989.02 | 2261.08 | 2963.04 |
|  | Constituent ratio | 8.85% | 6.01% | 9.09% | 8.93% | 5.54% | 4.91% |
| Diagnostic Fee | Sub-average  (yuan) | 1204.58 | 1618.16 | 1343.69 | 1146.12 | 1383.5 | 2114.7 |
|  | Constituent ratio | 4.09% | 4.00% | 4.24% | 3.42% | 3.39% | 3.50% |
| Laboratory fee | Sub-average  (yuan) | 1033.52 | 2152.6 | 1390.93 | 1202.29 | 1501.69 | 2485.05 |
|  | Constituent ratio | 3.51% | 5.32% | 4.39% | 3.59% | 3.68% | 4.12% |
| Chinese patent medicine fee | Sub-average  (yuan) | 605.69 | 461.34 | 391.38 | 461.14 | 179.66 | 756.52 |
|  | Constituent ratio | 2.06% | 1.14% | 1.24% | 1.38% | 0.44% | 1.25% |
| Other fees | Sub-average  (yuan) | 340.75 | 1749.3 | 543.91 | 188.9 | 487.55 | 2532.06 |
|  | Constituent ratio | 1.16% | 4.32% | 1.72% | 0.56% | 1.19% | 4.19% |
| Sanitary materials fee | Sub-average (yuan) | 28.7 | 54.11 | 46.47 | 23.36 | 44.98 | 51.81 |
|  | Constituent ratio | 0.10% | 0.13% | 0.15% | 0.07% | 0.11% | 0.09% |
| Total | | 29532.92 | 40455.91 | 31658.88 | 33482.28 | 40850.42 | 60369.29 |
